# Ileosigmoid Anastomotic Perforation Three Weeks After Placement of Lumen-Apposing Metal Stent (LAMS)

**DOI:** 10.7759/cureus.20565

**Published:** 2021-12-21

**Authors:** Nader Mekheal, Harry Aslanian, Vivek Kesar, Priya Jamidar, Thiruvengadam Muniraj

**Affiliations:** 1 Internal Medicine, St. Joseph's Regional Medical Center, Paterson, USA; 2 Gastroenterology, Yale School of Medicine, New Haven, USA; 3 Gastroenterology, Yale New Haven Hospital, New Haven, USA; 4 Gastroenterology, Yale University, New Haven, USA

**Keywords:** lumen-apposing stent, lams, perforation, gastrointestinal stricture, git endoscopy

## Abstract

Benign anastomotic intestinal strictures are difficult to manage as there may be limited response to dilation. Fully covered self-expanding metal stents have been utilized in small case series; however, stent suturing is required due to the high risk of stent migration. Lumen-apposing metal stents (LAMS) are fully covered and have a novel dumbbell shape that prevents stent migration. Initial reports identify low migration rates and good clinical success rates. This is the first report of perforation following treatment of an ileosigmoid stricture in a 52-year-old female, three weeks after LAMS placement.

## Introduction

Lumen-apposing metal stents (LAMS) are fully covered dumb-bell shape stents with flanged ends that were originally designed to drain pancreatic fluid collection [1]. Intestinal anastomotic strictures are often difficult to manage as they may be refractory to dilation. Fully covered self-expanding metal stents (SEMS) have been utilized to treat upper intestinal strictures; however, the covering which prevents permanent embedding in the intestinal wall leads to a high migration rate and suturing of the stent followed by removal within a few months is required. The enlarged dumb-bell like proximal and distal flanges of LAMS prevent stent migration and initial case series have reported good clinical success for benign strictures [1-5]. Adverse events include pain, bleeding, ulceration and recurrence of the stricture after removal [6-8]. We present a case of perforation in a patient three weeks after the placement of a 15x10 mm LAMS (Axios, Boston Scientific, Marlborough, MA, United States) for the treatment of an ileosigmoid anastomotic stricture [1, 6].

## Case presentation

**Figure 1:**
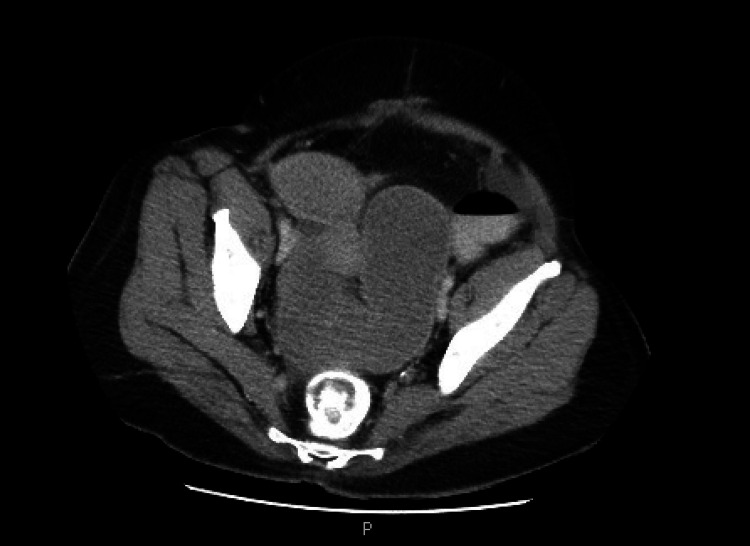
Computed tomography of the abdomen showing worsening high-grade small bowel obstruction

**Figure 2:**
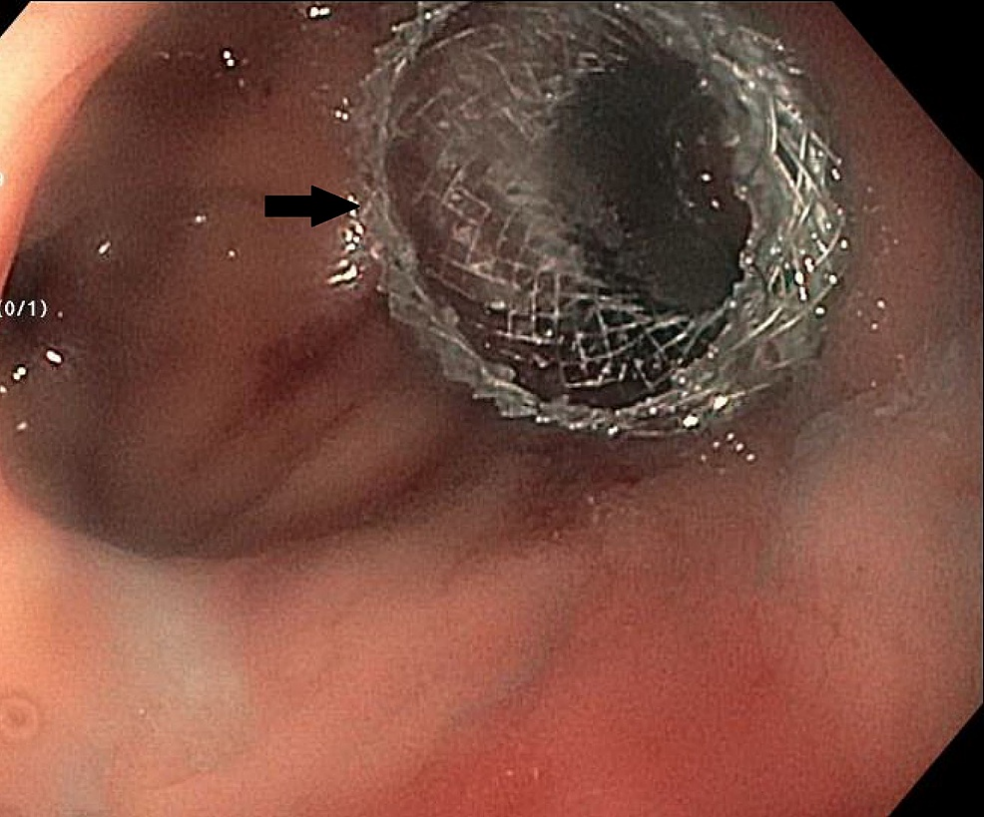
Endoscopic image of the ileosigmoid stricture after deployment of the AXIOS stent (arrow)

**Figure 3:**
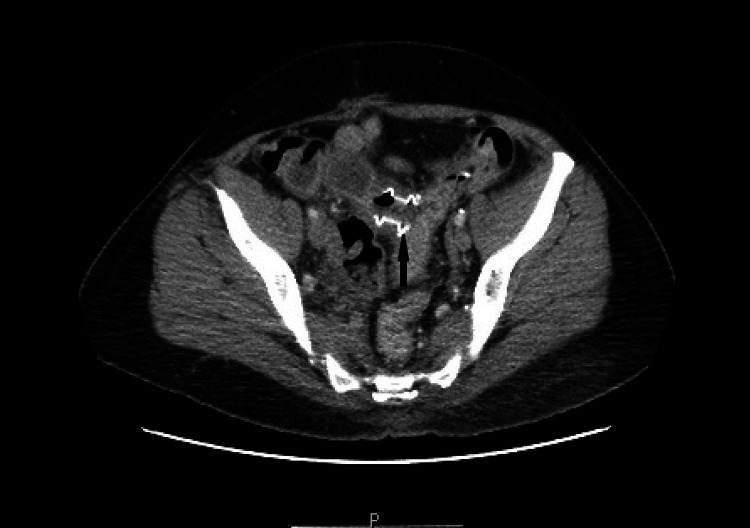
Computed tomography of the abdomen showing the AXIOS stent (arrow) at the ileosigmoid stricture relieving the obstruction

A 52-year-old female had a past medical history of ischemic pancolitis with transmural necrosis of the splenic flexure, status post subtotal colectomy with end ileostomy and subsequent takedown with ileosigmoid anastomosis. She presented with abdominal pain, nausea and vomiting. CT of the abdomen showed small bowel obstruction at the ileosigmoid anastomosis level (Figure 1). Flexible sigmoidoscopy identified a short, 3 mm length anastomotic stricture. The stricture was dilated to 12 mm using a wire-guided balloon (CRE, Boston Scientific, Marlborough, MA, United States) to reduce the risk of trauma and perforation. Under fluoroscopic and endoscopic guidance, a 15 x 10 mm LAMS was deployed across the stricture which resulted in drainage of a large amount of stool (Figures 2, 3). The patient’s symptoms of obstruction resolved and stent removal was planned in two to three months.

**Figure 4:**
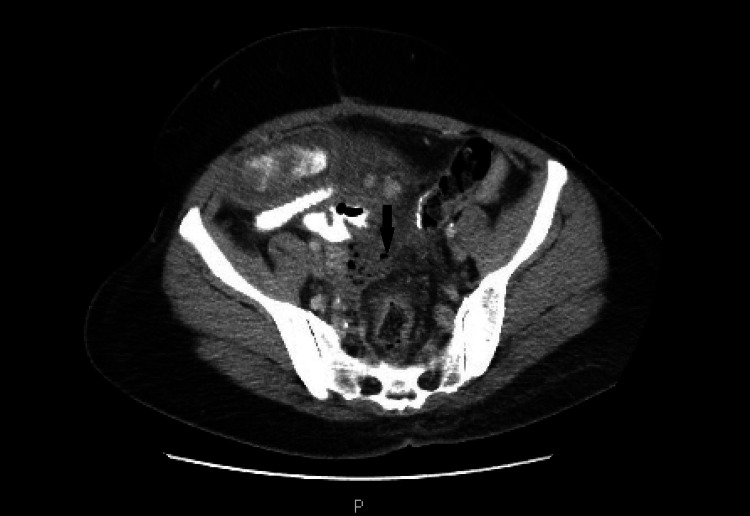
Computed tomography of the abdomen showing dilatation and wall thickening of a long segment of distal small bowel in the right anterior hemiabdomen with multiple surrounding foci of intraperitoneal air (arrow)

Three weeks later, the patient presented to the emergency room with severe abdominal pain. CT of the abdomen showed small bowel obstruction with multiple foci of intraperitoneal air concerning for perforation (Figure 4) and a 6 x 2.1 cm fluid collection concerning for ileosigmoid anastomotic breakdown. Operative exploration identified perforation at the level of the ileosigmoid anastomosis with purulent fluid and fibrous adhesions. The ileosigmoid anastomosis was transected, and an end ileostomy was created. The patient was discharged one week later in stable condition.

## Discussion

Anastomotic strictures often present a management challenge. Endoscopic balloon dilation with or without steroid injection is typically the first-line therapy for gastrointestinal (GI) anastomotic strictures. Temporary placement of fully covered SEMS has been utilized for the treatment of upper intestinal tract anastomotic strictures; however, endoscopic suturing of the stent to the intestinal wall to prevent stent migration is required [2]. Frequent adverse events with fully covered SEMS include stent migration and stricture recurrence after stent removal. The novel dumb-bell shape of LAMS reduces the risk of migration and the initial series shows promising results for off-label use in the treatment of GI strictures [2].

Yang et al identified a clinical success rate of 64% in a retrospective study of 30 patients treated with LAMS for benign gastrointestinal strictures [5]. Irani et al reported a clinical success rate of 82.9% in 25 patients with benign strictures treated with LAMS [5-6]. Both studies identified a migration rate of 7-8% with LAMS compared to 15-33% reported with fully covered SEMS, with variance based on the technique used to secure the stent (over-the-scope clip vs. suturing) [3-4]. Another retrospective study of 18 patients, with persistent gastrojejunal anastomosis stenosis status post gastric bypass surgery, showed a technical success rate of 100% and a clinical success rate of 94% after placing LAMS [7,8]. Lastly, in one multicenter series of 49 patients with LAMS utilized for benign strictures, technical success and clinical success rates were 100% and 96.4% respectively with 4% of patients developing abdominal pain requiring stent removal. In this study, stent migration occurred in 17.9% of patients with the lower gastrointestinal tract being the most common site [8-9].

Although LAMS placements are well-tolerated procedures, they are not free of complications. Adverse events with LAMS included abdominal pain with subsequent ulceration identified at the time of stent removal from the site of gastrojejunal anastomotic strictures, in two of 25 patients, one to three weeks after stent placement [6]. In another series, one of 21 patients with benign strictures managed with LAMS, developed pain and ulceration 28 days after placement, requiring immediate removal [10].

Additional adverse events reported in extensive review analysis of the literature available until 2017 were bleeding, vomiting, stent migration, death, and formation of new strictures proximal to the LAMS. In this review, they also included one study in which a perforation happened immediately after the procedure requiring surgery that they considered a technical failure [1]. Another case in which the distal LAMS flange perforated through the anterior duodenal wall two weeks after insertion across a malignant pyloric stricture has recently been reported [11]. Here we present another case of LAMS perforation of a benign stricture and of a stricture in the lower intestinal tract highlighting that perforation is a potentially rare but known complication associated with LAMS. While the novel shape of fully covered LAMS provides a much-needed therapeutic potential for benign gastrointestinal strictures additional evaluation of adverse events, including ulceration and perforation, is required to consider the optimal design of the stent for this indication.

## Conclusions

Managing anastomotic strictures often present a great challenge. Endoscopic balloon dilation with or without steroid injection is first-line therapy. Temporary placing fully covered SEMS is another option; however, has its own limitations and complications. On the other hand, lumen-apposing metal stents (LAMS) provide another approach to manage gastrointestinal strictures. It has great therapeutic potential with its novel dumbbell shape; however, ulceration and perforation are major concerns that require further consideration of the optimal design of the stent design for this indication.
